# RAISING OUR VOICES: THE BENEFITS OF AN INTERGENERATIONAL CHOIR FOR PEOPLE WITH DEMENTIA AND THEIR CARE PARTNERS

**DOI:** 10.1093/geroni/igz038.2758

**Published:** 2019-11-08

**Authors:** Debra J Sheets, Theresa A Allison

**Affiliations:** 1 University of Victoria, Victoria, British Columbia, Canada; 2 University of California, San Francisco, San Francisco, California, United States

## Abstract

This interdisciplinary symposium focuses on the Voices in Motion (ViM) choir, a novel social intervention to address issues of stigma and social isolation among older adults with dementia and their caregivers. ViM is an intergenerational choir for community-dwelling older adults with dementia (PwD) and their caregivers. Local high school students participated in the choir and added to the lively social interactions. Two professionally directed ViM choirs were fully implemented in 2018-2019 with a public performance in the Fall and Spring seasons. This symposium brings together multiple methodologies to investigate the effects of choir participation on cognition, social connections, stigma, and quality of life for the dyads. Results in the individual papers demonstrate the positive impact of choir participation on dyads (n=26) for measures that includecognition (MacDonald), well-being and quality of life (Sheets), and social connections (Smith). Taken as a whole, the papers indicate that this social intervention offers an effective non- pharmacological alternative approach for older adults with dementia. Choir participation has important and significant impacts on psycho-social well-being and quality of life. The body of evidence presented points to the importance of intergenerational programs that are dementia-friendly and that support meaningful participation by older adults with dementia in the broader community. Discussion focuses on implications for social policy with attention on the replication and sustainability of the program.

